# Spotlight on… Gabriella Campadelli-Fiume

**DOI:** 10.1093/femsle/fny019

**Published:** 2018-05-23

**Authors:** Gabriella Campadelli-Fiume

**Affiliations:** Department of Experimental, Diagnostic and Specialty Medicine, University of Bologna, Via San Giacomo, 12 40126 Bologna, Italy

## Biographical Summary

My first education was in biochemical pathogenesis of disease at the University of Bologna, Italy, University College, London and Max Planck institute, Heidelberg. I entered Virology in 1972 in the University of Bologna, where I still work. I am the head of the molecular virology laboratory in the Department of Experimental Medicine, University of Bologna. The focus of my research is on the molecular mechanisms of herpes simplex virus (HSV) entry into the cell, and on how to modify the HSV tropism in order to generate highly cancer-specific, highly safe, yet fully virulent oncolytic herpesviruses. Academically, I fostered the integration of the Italian Virology in the European Virology.

**Figure:**
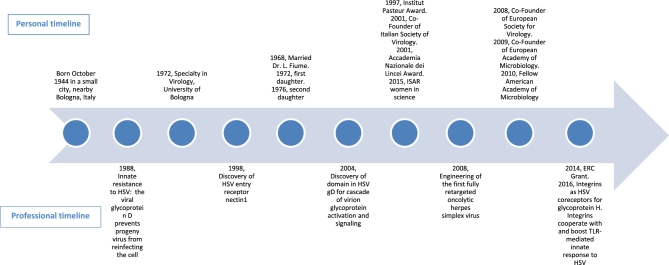


## A) CONCISE WRITTEN ANSWERS TO EACH OF THE FOLLOWING QUESTIONS:

### 1. What is your current research addressing and what impact may this research have on the wider field?

The entry of herpes simplex virus (HSV) into the cell has been my major field of interest for +20 years (Campadelli-Fiume *et al.*^1990^; Brandimarti *et al.*^1994^; Cocchi *et al*. 1998a,b; Campadelli-Fiume *et al.*^2000^; Campadelli-Fiume *et al.*^2007^).This process entails a bunch of virion glycoproteins that are activated in a cascade manner by interaction with a number of cognate cellular receptors and intermolecular signaling (Menotti *et al.*^2000^; Cocchi *et al.*^2004^; Fusco, Forghieri and Campadelli-Fiume 2005; Gianni, Gatta and Campadelli-Fiume 2010; Gianni, Leoni and Chesnokova 2012; Gianni and Campadelli-Fiume 2014; Gianni, Massaro and Campadelli-Fiume 2015). It has been a competitive area. On this background, some 10 years ago I decided to enter the field of oncolytic viruses and design highly cancer-specific oncolytic herpes simplex viruses (oHSVs), by re-directing the virus tropism to cancer receptors of choice and abolishing the virus tropism for its natural receptors (Menotti *et al.*^2008^; Menotti *et al.*^2009^; Campadelli-Fiume *et al.*^2011^; Nanni *et al.*^2013^). Through this approach, we also improved the current understanding of the biology of HSV entry. Currently, my aim is to translate the results of preclinical studies from my lab into clinical experimentation. Most likely within the foreseeable future, the retargeted oHSVs will have clinical applications as fully virulent highly safe onco-immunotherapeutic HSVs.

### 2. What made you decide on a career in Microbiology?

I entered Virology by serendipity. My husband—a faculty member at the University of Bologna—and I spent 2 years in European scientific institutions, and then returned to Italy. I wanted to do research, but this was a rather vague aspiration. I had no trust in myself, and had no idea what kind of researcher I could or wanted to be. The easiest path was to search for a position at the University of Bologna. I joined a lab headed by a scientist who had just made a move to Medical Virology, a burgeoning field in the 70s. Since there was no PhD program in Italy at that time, I took a residency in Virology. Basically, my initial work entailed jumping from one virus to the other, and what I learnt was basic virology. A few years later, I had a chance to join the late Maurizio Terni, who introduced me to herpesvirology and to Bernard Roizman. After a few years, I decided to move into the field of HSV glycoproteins and HSV entry into cells. This was the arena in which I played my game as a tiny sand granule.

### 3. Who or what had the most positive influence on your career? Who is your microbiology hero/heroine (living or dead) and why?

Two scientists made a major impact on my scientific life. The first was my husband, Luigi Fiume. By virtue of his own example, he taught me intellectual honesty and integrity, to be a dedicated scientist, to distinguish between ambition and vanity, not to be flattered by the glitter of science. Yet, I did not acquire self-awareness. The second was Bernard Roizman, considered to be the father of herpesvirology. I met him when I was in my mid 30’s, after my second daughter was born. Remarkably, he did not care much that I worked in an obscure laboratory in a scientifically non-prominent country, or that I had not had solid training except self-teaching. He was confident that I could become a good scientist, prompted me to take a genetic approach to HSV and invited me to his lab, a place reputed to be the Hallowed Halls of Herpesvirology. I built my self-awareness slowly, thanks to his trust in me. I am grateful to both.

At the twilight of my career, Alfredo Nicosia and the late Riccardo Cortese – two extraordinary researchers – made a significant impact on me. They decided to take the retargeted oHSVs I designed to the translational phase, and asked me to join them. In a way, I was too old to fully rewire myself and to proficiently take profit of such an amazing opportunity. Yet, they managed to introduce me to the translational medical science. I am deeply grateful.

### 4. What do you consider to be the most important skills for a microbiologist?

As for any researcher, the most important skill for a microbiologist is the ability to ask a ‘good question.’ The question needs to address a key topic, at the heart of a relevant, open and unresolved issue. The second skill has to do with method in science. When one looks at results, one should take an unbiased view, forget the hypothesis that prompted the experiment and look at data at face value without prejudice. Curiosity is our motivation and motor. New questions and progress in science often arise from the realization of the importance of the actual data that may seem anomalous because they do not fit with the expected results.

### 5. What advice would you offer to early career researchers in microbiology to help further their career? What is the best advice you can give for maintaining the work–life balance?

I would like to offer my advice to women scientists. For most of us, both women and men, science is not our whole life. Family, children, a companion and intellectual activities are all fundamental parts of life. Combining science and personal life is not an easy task. Women tend to be less self-confident and less assertive than men. Pregnancy and child-rearing require dedication, energy and time. Thus, biology and tradition conspire to make these activities largely female duties. In this vicious circle, quite too often and too easily, women scientists subordinate the pursuit of a scientific carrier to their personal life. The advice is not to give up science, and to be assertive. Be aware of Virginia Woolf's lesson in A Room of One's Own.

### 6. What would you say is the greatest challenge facing microbiologists today?

 In my view, the worldwide impact of basic virology has reached its peak. Key open questions include the challenges raised by the human viruses that cause persistent infections—human immunodeficiency virus, hepatitic C virus and herpesviruses—including our limited ability to design strong therapeutic vaccines, the emerging and reemerging viruses in Africa, South America, Middle East, the plant viruses that infect crops. The basic molecular biology of bacteria is now in its renaissance. Multi-drug resistant bacteria represent the new menace, and microbiologists will have to find ways to deal with it. The war of a microbe to its host, and the counteractions that the host has evolved to control microbes are and will remain at the heart of microbiology, and its applications.

## B) A VERY BRIEF SUMMARISING COMMENTARY OF YOUR OVERALL EXPERIENCE AS A MICROBIOLOGIST.

 I worked during years in which virology developed as a basic science. I had the chance to witness its advances from a phenomenological description of events, to a quantitative science, to the genetics of virus replication and finally to the molecular and structural basis of the virus–cell interaction. I met bright scientists, and made some very good long-term friends. I did not expect much of myself, and did not expect such an amazing and intellectually rewarding life when I entered virology.

## C) A RECENT PHOTOGRAPH OF YOURSELF.

### D) A BRIEF BIOGRAPHICAL SUMMARY (100 WORDS INCLUDING CURRENT POSITION/AFFILIATION/MAIN FOCUS OF ACTIVITY) TO APPEAR ALONGSIDE THE PHOTOGRAPH.

Except for a few years at the University of Milano, and a few years in Europe and at the University of Chicago, for most of my life I have been working in the University of Bologna, Italy, where I still work. Here, I served as a Professor of Microbiology and Virology in the Biotechnology School, and in the Medical School. Although very recently I formally retired, I still keep my lab thanks to an European Research Council Advanced (ERC AdG) Grant.

## E) A PERSONAL AND PROFESSIONAL TIMELINE (USING THE ATTACHED MATRIX OR A SIMPLE TEXT BOX), AND PLEASE FEEL FREE TO NOT ENTER CERTAIN DATES (I.E. ONLY PROVIDE A RELATIVE TIMESCALE).
